# Correlation of mutational landscape and survival outcome of peripheral T-cell lymphomas

**DOI:** 10.1186/s40164-021-00200-x

**Published:** 2021-02-05

**Authors:** Yingying Ye, Ning Ding, Lan Mi, Yunfei Shi, Weiping Liu, Yuqin Song, Shaokun Shu, Jun Zhu

**Affiliations:** 1 Key Laboratory of Carcinogenesis and Translational Research (Ministry of Education), Department of Lymphoma, Peking University Cancer Hospital & Institute, Haidian District, No 52, Fucheng Road, Beijing, 100142 China; 2 Key Laboratory of Carcinogenesis and Translational Research (Ministry of Education), Department of Pathology, Peking University Cancer Hospital & Institute, Haidian District, No 52, Fucheng Road, Beijing, 100142 China; 3 Department of Biomedical Engineering, Peking University, Beijing, 100871 China

**Keywords:** Peripheral T-cell lymphomas, Ten-eleven translocation 2, Protein 53, Prognosis

## Abstract

**Objective:**

To explore the correlation of mutation landscape with clinical outcomes in patients with peripheral T-cell lymphoma (PTCL).

**Methods:**

We retrospectively analyzed the clinicopathological and prognosis data of 53 patients with PTCL from November 2011 to December 2017. Targeted next-generation sequencing of a 659-gene panel was performed for tissues from 53 patients with PTCLs. The correlation of mutation landscape with clinical outcomes was analyzed.

**Results:**

*TET2* was the most frequently mutated gene (64%), followed by *RHOA* (43%), *PCLO* (23%), *DNMT3A* (19%), *IDH2* (17%), *PIEZO1* (17%) and *TP53* (15%). When mutated genes were categorized into functional groups, the most common mutations were those involved in epigenetic/chromatin modification (75%), T-cell activation (74%), and the DNA repair/TP53 pathway (64%). *TET2/TP53* mutations were significantly associated with positive B symptoms (*P* = 0.045), and elevated lactate dehydrogenase (LDH) level (*P* = 0.011). Moreover, *TET2/TP53* mutation was a risk factor for PTCL patient survival (HR 3.574, 95% CI 1.069 − 11.941,* P* = 0.039). The occurrence of JAK/STAT pathway mutations in angioimmunoblastic T-cell lymphoma (AITL) patients conferred a worse progression-free survival (HR 2.366, 95% CI 0.9130–6.129, *P* = 0.0334).

**Conclusions:**

Heterogeneous gene mutations occur in PTCL, some of which have a negative impact on the survival outcome.

## Background

Peripheral T-cell lymphoma (PTCL) is a group of heterogeneous clinicopathological entities of non-Hodgkin lymphoma (NHL) and is often characterized by its aggressive disease course and poor clinical outcome, representing 10% to 15% of all lymphoid malignancies [1]. Recent high-throughput genomic and molecular profiling researches have provided a great deal of information that has prominently improved our understanding of the pathogenesis and pathobiology of these malignant diseases. Mutations within PTCLs recurrently occur in different classes of epigenetic modifiers [2] including ten-eleven translocation 2 (*TET2*) [3], isocitrate dehydrogenase 1/2 (*IDH1/2*) [4], and DNA methyltransferase 3A (*DNMT3A*) [5], as well as in the T-cell receptor (TCR) and coreceptor signaling pathways [6], and components or regulators of the JAK/STAT signaling pathway [7]. Although multiple genetic alterations are shared among different PTCL subtypes, the frequency is significantly different and certain alterations are unique to a specific subtype. For example, *RHOA* and *TET2* mutations are frequently detected in angioimmunoblastic T-cell lymphoma (AITL) [8, 9] and *IDH2*^*R172*^ variant appears to be a unique mutation in AITL [4].

Given the limited value of chemotherapy for PTCLs, the clinical need of improving the treatment and outcomes of PTCL is still unmet. Better description of the mutational landscape may help advance our understanding of the value of the existing treatment options and development of novel therapeutic approaches. We hypothesized that differences may exist in the baseline clinical features and prognosis between different subgroups of patients according to their mutation spectrum. Therefore, to further explore the mutation profile of PTCL patients and correlate the molecular spectrum with baseline clinical characteristics and outcomes, we retrospectively applied targeted sequencing analysis of patients with PTCLs.

## Methods

### Patients and samples

A total of 53 patients with PTCLs diagnosed in Peking University Cancer Hospital (China) between November 2011 and December 2017 were enrolled for next-generation sequencing. Among these patients, 49 with sufficient clinical information were included in the clinical relevance analysis. The diagnosis of PTCL was based on the criteria of the 2008 World Health Organization (WHO) classification [10]. Tumor tissue biopsies were collected from the patients before the initial therapy and guardians of all the patients provided informed consent in accordance with the Declaration of Helsinki. Medical records were reviewed for clinical and pathological data. All procedures were approved by the Institutional Review Board and the Ethical Committee of Peking University Cancer Hospital and Institute.

### DNA extraction

The genomic DNA (gDNA) of formalin-fixed, paraffin-embedded (FFPE) samples and fresh-frozen tissue samples was isolated by using the Maxwell® 16 FFPE Plus LEV DNA Purification Kit (Qiagen, Hilden, Germany; catalog: AS1135) and the DNeasy Blood & Tissue Kit (Qiagen, Hilden, Germany), respectively. The DNA concentration was quantified using a Qubit fluorometer and the Qubit dsDNA HS (High Sensitivity) Assay Kit (Invitrogen, Carlsbad, CA, USA).

### Library preparation and target enrichment

One μg each of genomic DNA sample was sheared into fragments of 300 bp in length by sonication (Covaris S2 ultrasonicator, Covaris, Woburn, MA, USA) before library construction. The KAPA DNA Library Preparation Kit (Kapa Biosystems, Wilmington, MA, USA) was used for the preparation of indexed Illumina next-generation sequencing (NGS) libraries of gDNA. A custom SeqCap EZ Library (Roche NimbleGen, Madison, WI, USA) was applied for target enrichment. To investigate the genetic properties of PTCL, we designed a capture probe basing on genomic regions of approximately 2.4 Mb from 659 genes (see Additional file 1: Table S1) that are frequently mutated in PTCLs and other common hematological malignancies. Barcoded libraries were hybridized according to the manufacturer’s protocol. DNA fragments captured from the hybrid selection were then amplified and combined to generate various libraries.

### Next-generation sequencing and sequence data analysis

The gDNA libraries were subjected to high-throughput sequencing with 150-bp pair-end reads on the NovaSeq 60,000 Sequencing System (Illumina, San Diego, CA) supported by a commercial vendor (Geneplus-Beijing, China). The average sequencing depth of tissues was 506× (136× to 1003×). Sequence reads were aligned using BWA version 0.5.9 (Broad Institute). Single nucleotide variants (SNVs) were called using MuTect (version 1.1.4) and NChot [11, 12]. Small insertions and deletions (Indels) were determined by GATK [13]. All final candidate variants were manually reviewed by using the IGV browser.

### Statistical analysis

Overall survival (OS) was considered from disease confirmation to the end of follow-up or death. The date of diagnosis to the date of disease progression, death or last follow-up was determined as progression-free survival (PFS). We performed group comparisons by using Fisher’s exact test and Student’s *t*-test for categorical and continuous variables, respectively. Survival was assessed by Kaplan–Meier and log-rank methods. The Cox regression model was adopted to evaluate the prognostic factors. Statistical analyses were performed with Graphpad Prism version 8.0.1 and SPSS version 26.0. *P* < 0.05 was considered statistically significant.

## Results

### Patient characteristics

All patients had histopathologically confirmed PTCLs, comprising angioimmunoblastic T-cell lymphoma (AITL; n = 33, 62%), peripheral T-cell lymphoma, not otherwise specified (PTCL-NOS; n = 10, 19%), anaplastic lymphoma kinase (ALK) positive anaplastic large cell lymphoma (ALK + ALCL; n = 4, 8%) and ALK negative ALCL (ALK − ALCL, n = 6, 11%). The general characteristics of the 49 PTCLs patients with sufficient clinical information for inclusion in the clinical relevance analysis are summarized in Table 1. The median age was 54 years (range 26–80 years). Most (34 of 49, 69%) patients were males and diagnosed at stage 3 or stage 4 (40 of 49, 82%), and 15 (31%) patients had international prognostic index (IPI) scores of 3 to 5. Elevated serum lactate dehydrogenase (LDH) was detected in 31 (63%) patients, and 24 (50%) patients presented with positive B symptoms.

**Table 1:** Patients' characteristics based on *TET2* and *TP53* mutational status

Clinical Parameters	Overall N (%)	TET2			TP53			TET2/TP53		
		WT N (%)	Mut N (%)	*P* value*	WT N (%)	Mut N (%)	*P* value*	WT N (%)	Mut N (%)	*P* value*
N	49	18 (37)	31 (63)		41 (84)	8(16)		14 (29)	35 (71)	
Gender										
Male	34 (69)	11 (32)	23 (68)	0.338	31 (91)	3 (9)	0.085	10 (29)	24 (71)	1.000
Female	15 (31)	7 (46)	8 (54)		10 (67)	5 (33)		4 (27)	11 (73)	
Age at diadnosis (years)										
≤ 60	35 (71)	13 (37)	22 (63)	0.925	28 (80)	7 (20)	0.501	10 (29)	25 (71)	1.000
> 60	14 (29)	5 (36)	9 (64)		13 (93)	1 (7)		4 (29)	10 (71)	
Diagnosis										
Angioimmunoblastic T-cell lymphoma	29 (59)	4 (14)	25 (86)	< 0.001	28 (97)	1 (3)	0.003	4 (14)	25 (86)	0.028
PTCL, not otherwise specified	10 (21)	5 (50)	5 (50)		7 (70)	3 (30)		4 (40)	6 (60)	
ALK+ anaplastic large cell lymphoma	4 (8)	3 (75)	1 (25)		3 (75)	1 (25)		3 (75)	1 (25)	
ALK− anaplastic large cell lymphoma	6 (12)	6 (100)	0 (0)		3 (50)	3 (50)		3 (50)	3 (50)	
B symptoms										
Negative	23 (49)	12 (52)	11 (48)	0.025	20 (87)	3 (13)	1.000	10 (44)	13 (56)	0.045
Positive	24 (51)	5 (21)	19 (79)		21 (88)	3 (12)		4 (17)	20 (83)	
LDH										
Negative	18 (37)	10 (56)	8 (44)	0.037	15 (83)	3 (17)	1.000	9 (50)	9 (50)	0.011
Positive	31 (63)	8 (26)	23 (74)		26 (84)	5 (16)		5 (16)	26 (84)	
ECOG										
0–1	44 (90)	16 (36)	28 (64)	1.000	36 (82)	8 (18)	0.169	12 (27)	32 (73)	1.000
2–3	5 (10)	2 (40)	3 (60)		5 (100)	0 (0)		2 (40)	3 (60)	
Ann Arbor stage at diagnosis										
I–II	9 (18)	8 (89)	1 (11)	0.001	6 (67)	3 (33)	0.304	5 (56)	4 (44)	0.115
III–IV	40 (82)	10 (25)	30 (75)		35 (88)	5 (12)		9 (23)	31 (77)	
IPI score										
0–2	34 (69)	12 (35)	22 (65)	0.753	27 (79)	7 (21)	0.426	9 (27)	25 (73)	0.883
3–5	15 (31)	6 (40)	9 (60)		14 (93)	1 (7)		5 (33)	10 (67)	
Ki67										
≤ 75%	35 (74)	10 (29)	25 (71)	0.133	32 (91)	3 (9)	0.029	9 (26)	26 (74)	0.892
> 75%	12 (26)	7 (58)	5 (42)		7 (58)	5 (42)		4 (33)	8 (67)	
Extra nodal site										
≤ 1	33 (67)	11 (33)	22 (67)	0.478	27 (82)	6 (18)	0.926	8 (24)	25 (76)	0.531
> 1	16 (33)	7 (44)	9 (56)		14 (87)	2 (13)		6 (37)	10 (63)	

*WT* Wild-type, *LDH* lactate dehydrogenase, *ECOG* Eastern Cooperative Oncology Group, *IPI* International Prognostic Index

### The mutational landscape of PTCL

**Fig. 1:**
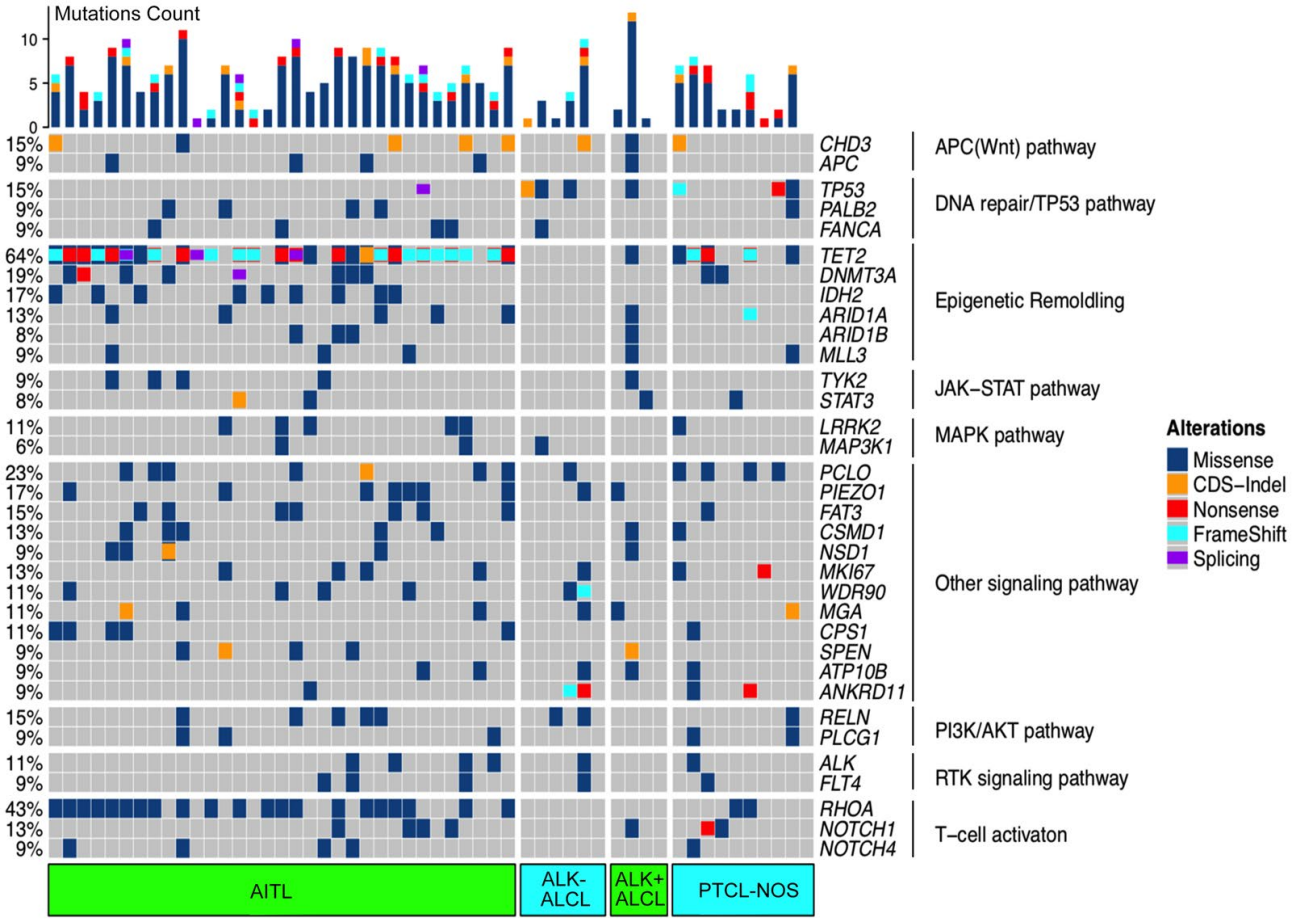
Gene mutation profile in 53 patients with newly diagnosed PTCL. Each column represents an individual patient. The block color represents the type of mutation. The percentages on the left of the table represent percent of study patients with a mutation in each gene. The bar graphs to the top of the plot indicate the number of mutations in each case

**Fig. 2:**
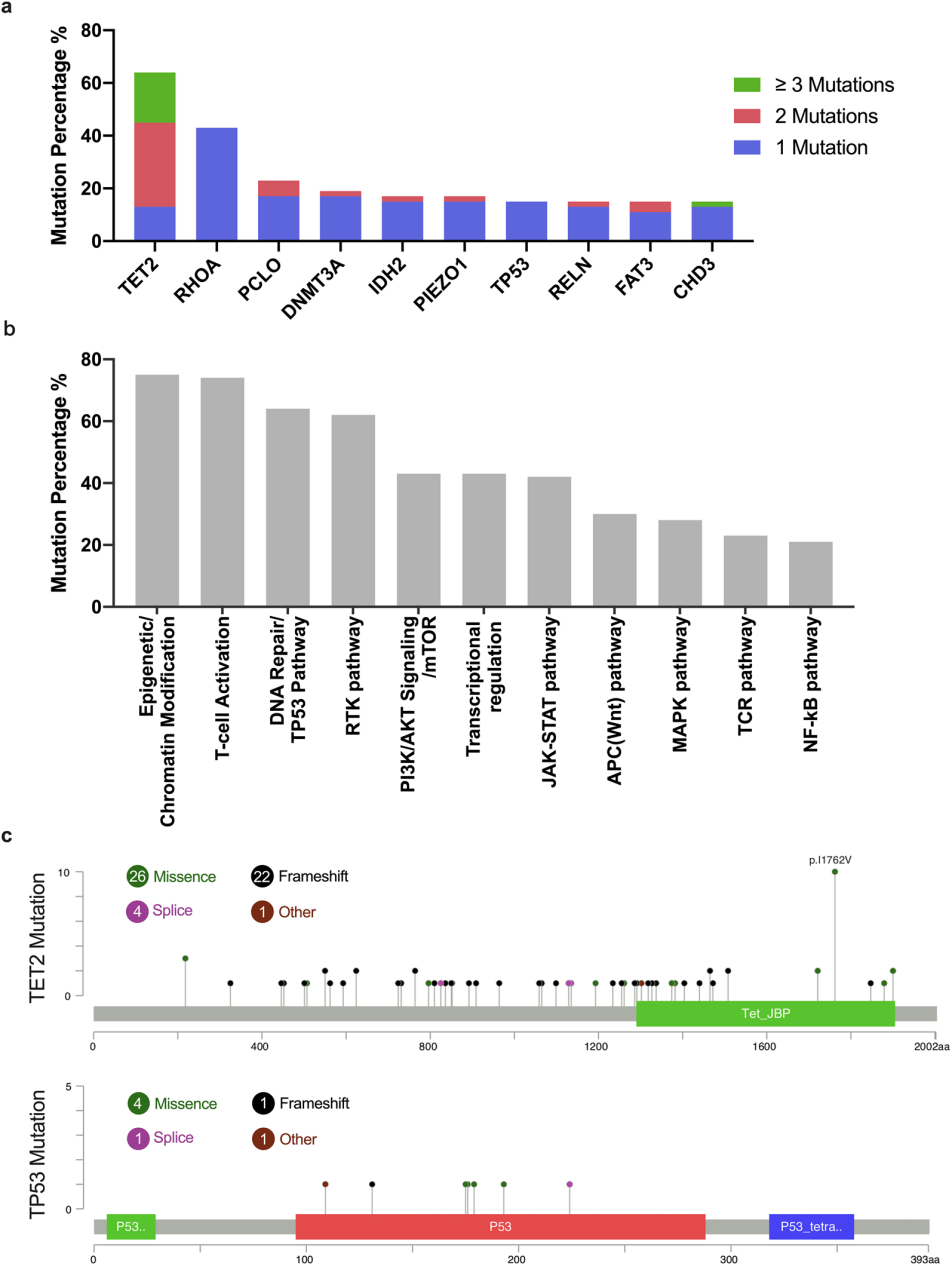
**a** Mutation percentage of the top ten frequently mutated genes in the cohort. **b** Gene mutation percentage of different signaling pathway. **c** Mutation sites of *TET2* and *TP53* in PTCL. Different colors indicate different types of mutations

We identified 856 non-dbSNP variants (750 SNVs and 106 Indels) within the coding regions of 334 genes, including 176 genes mutated in ≥ 2 cases (see Additional file 2: Table S2). In addition, mutations in 10 genes (including driver genes such as *TET2, RHOA, DNMT3A, IDH2,* and *TP53*, which are well-known PTCL hotspot panels) were found in the specimens of more than 15% patients (Fig. 1, 2a). The most frequent mutation in the whole cohort was observed in *TET2* (n = 34, 64%; including 17 with double mutations and 10 with ≥ 3 mutations), followed by *RHOA* (n = 23, 43%), *PCLO* (n = 12, 23%; including 3 double mutations) and *DNMT3A* (n = 10, 19%, including 1 double mutation) (Fig. 2a). In our study, a total of 15 *PCLO* mutations were observed in 12 patients, comprising seven AITL patients (21%), four PTCL-NOS patients (40%) and one ALK- ALCL patient (17%) (Fig. 1). Heterogeneous genetic mutations were detected in different histological types. The most frequent mutation was *TET2* (85%) in AITL patients, *PCLO* (40%) in PTCL-NOS patients, *TP53* (25%) in ALK + ALCL patients, and *TP53* (50%) in ALK- ALCL patients (Fig. 1). We further identified the distribution of gene mutations in different histological subtypes. As shown in Additional file 3: Fig. S1a, *TET2, RHOA* and *DNMT3A* mutations tended to cluster in AITL and PTCL-NOS patients, while none in ALK + or ALK- ALCL. And *IDH2* showed a subtype-specific mutation pattern, which only observed in AITL patients (27.2%). Mutations in *PCLO* were enriched in PTCL-NOS (40%), but *TP53* mutations observed at a relatively higher frequency in ALK- ALCL (50%).

When mutated genes were categorized into functional groups, the most common mutations were those involved in epigenetic/chromatin modifications (n = 40, 75%), T-cell activation (n = 39, 74%), the DNA repair/TP53 pathway (n = 34, 64%), the RTK pathway (n = 33, 62%), the PI3K/AKT signaling/mTOR pathways (n = 23, 43%), transcriptional regulation (n = 23, 43%), the JAK-STAT pathway (n = 22, 42%), the APC (Wnt) pathway (n = 16, 30%), the MAPK pathway (n = 15, 28%), the TCR pathway (n = 12, 23%), and the NF-κB pathway (n = 11, 21%) (Fig. 1, 2b). Mutations in epigenetic pathway and T-cell activation were mainly observed in AITL and PTCL-NOS, while mutated genes of DNA repair/ TP53 pathway in AITL and ALK- ALCL (Additional file 3: Fig. S1b).

In our study, *TET2* mutations were frequently detected in 28 (85%) AITL patients. Of note, 22 of the 28 AITL patients harbored more than two *TET2* mutations. Five of the 10 (50%) PTCL-NOS patients also had *TET2* mutations. The 75 *TET2* mutations comprised 26 missense mutations, 22 nonsense mutations, 22 frameshifts, four splices and one coding sequence insertion-deletion (CDS-Indel) (Additional file 3: Fig. S1c). *TP53* mutations occurred mainly in ALK- ALCL patients (n = 3, 50%), followed by PCTL-NOS patients (n = 3, 30%), ALK + ALCL patients (n = 1, 25%), and AITL patients (n = 1, 3%), comprising four missense mutations, one nonsense mutation, one frameshift, one splice and one CDS-Indel (Additional file 3: Fig. S1d). We further surveyed mutation sites in *TET2* and *TP53* in mutated samples. The *TET2* mutations were scattered throughout the genes, while *TP53* mutations were present mainly in the DNA binding domain (Fig. 2c).

### Clinical implications of gene mutations

We analyzed the clinical influence of the most common recurrent mutations (Table 1 and Additional file 4: Table S3). There were significant differences in the mutational status of *TET2*, *RHOA*, *IDH2*, and *TP53* among the pathological subgroups, and AITL patients were more likely to harbor mutations in *TET2* (88%; *P* < 0.001), *RHOA* (65%, *P* = 0.001), and *IDH2* (28%; *P* = 0.024) than in other types of PTCLs. We also observed correlations between several laboratory variables and the presence of *TET2* or *TP53* mutations (Table 1). Patients with *TET2* mutations tended to have positive B symptoms (*P* = 0.025), elevated LDH level (*P* = 0.037) and advanced disease (*P* = 0.001), while PTCL patients with *TP53* mutations were more likely to presented with higher Ki-67 expression (*P* = 0.029). *TET2/TP53* mutations were significantly associated with positive B symptoms (*P* = 0.045) and elevated LDH level (*P* = 0.011).

### Impact of mutations on clinical outcome

In our cohort, 46 patients received CHOP-like regimens in the frontline, and we analyzed the association between genetic mutations and clinical outcome. Among the 46 patients evaluated for response to CHOP-like therapy, 37 patients received the CHOP plus etoposide regimen, while the other nine patients received CHOP alone therapy. Seven patients who responded to CHOP-like treatment received autologous stem cell transplant (ASCT) consolidation therapy. And 9 patients were administrated with epigenetic therapy. The overall response (OR) rate of the 46 patients was 65.2% [30 of 46 patients], with 54.3% [25 of 46] patients achieving complete response (CR) and 10.9% (5 of 46) patients achieving partial response (PR). However, as shown in Additional file 5: Table S4, there was no correlation between the mutations identified in this study and the efficacy of CHOP-like chemotherapy.

**Fig. 3:**
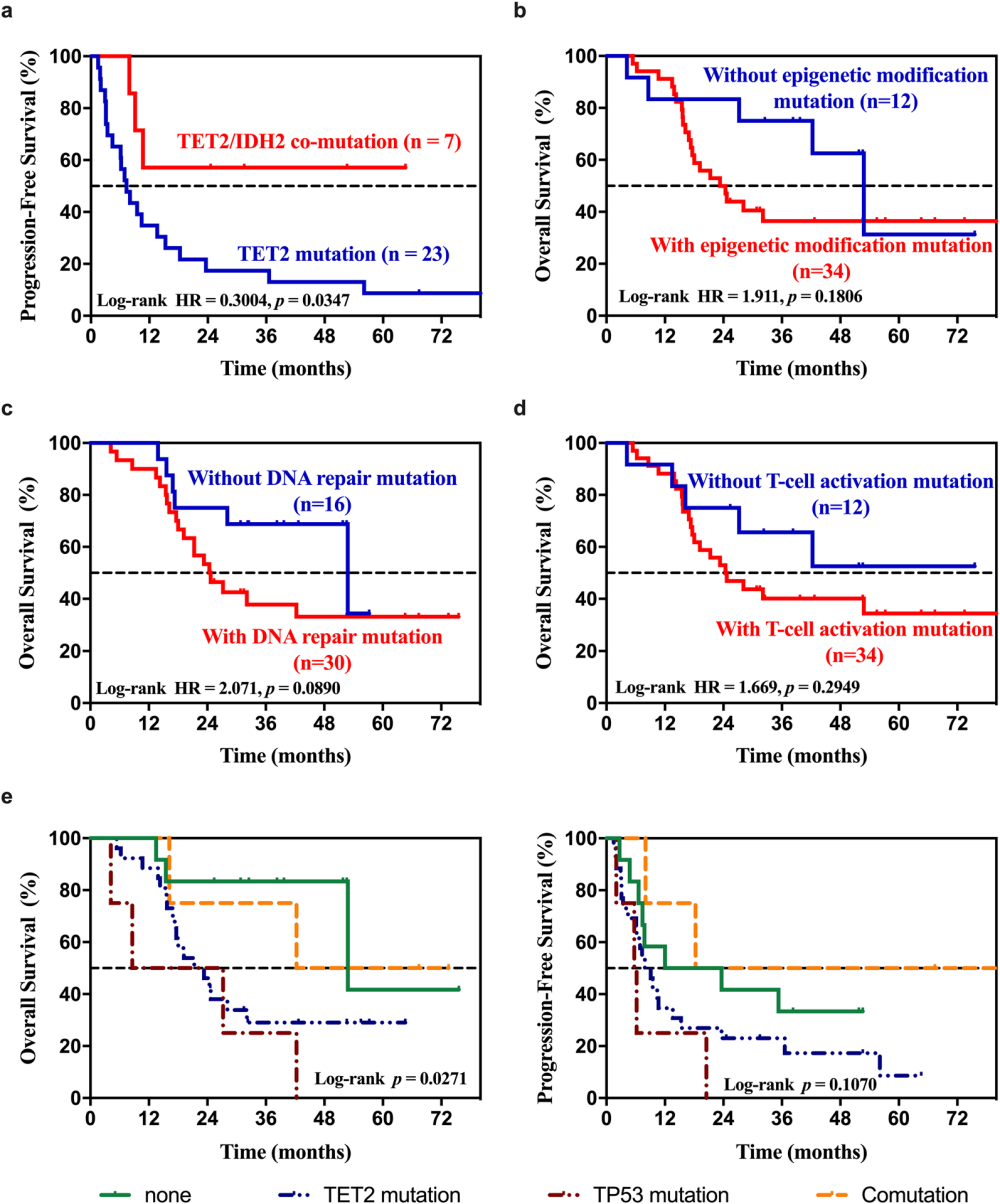
Kaplan–Meier curves for univariate analysis. Stratified by **a** *TET2, IDH2* co-mutation vs *TET2* mutation alone, **b** epigenetic modification mutation, **c** DNA repair pathway mutation, **d** T-cell activation mutation, **e** *TET2*, *TP53* mutation alone and co-mutation

The median follow-up time was 28.0 months (range 5.0–84.9 months). Thirty-seven (80.4%) patients progressed or relapsed and 26 (56.5%) patients died. The median OS was 26.3 months (range 4.2–85.2 months). The 3-year OS and PFS rates for the cohort were 46.9% and 25.7%, respectively. Patients with ASCT consolidation therapy tended to have a better OS (HR 0.1532, 95%CI 0.059–0.396, *P* = 0.0329, Additional file 6: Fig. S2a). No significant correlation between individual mutations and the OS of the cohort was observed. Patients carrying *TET2* and *IDH2* co-mutations showed a better PFS compared with only *TET2* mutated group (HR 0.3004, 95% CI 0.129–0.700, *P* = 0.0347, Fig. 3a).

We further analyzed the prognostic effect of epigenetic modification, T-cell activation and DNA repair pathway mutation in PTCL patients and found no significant differences in OS or PFS between the wild-type (WT) group and the mutated group (*P* > 0.05; Fig. 3b–d). However, patients with DNA repair pathway mutations demonstrated worse survival. The combination of *TET2* and *TP53* mutations provided much better prognostic capacity for distinguishing patients with a poor prognosis from those with a good prognosis (23.9 months vs. 52.8 months, HR 3.535, 95 CI 1.546–8.083, *P* = 0.0272, Fig. 3d). Patients with *TET2* and *TP53* co-mutations showed a superior OS compared with those with *TP53* mutations alone. In the univariate analysis, adverse prognosis factors were abnormal LDH level (HR 2.703, 95% CI 1.079–6.772, *P* = 0.034), and *TET2/TP53* mutations (HR 3.535, 95 CI 1.546–8.083, *P* = 0.0272). Multivariate analysis showed that *TET2/TP53* mutations (HR 3.574 95% CI 1.069 − 11.941, *P* = 0.039) was an independent adverse risk factor for OS (Table 2).

**Table 2:** Influence of clinical parameters and gene mutations on OS

	Univariate analysis ^a^			Multivariate analysis ^b^		
	HR	95%CI	*P* Value*	HR	95%CI	*P* Value*
Age ≥ 60	2.141	0.946–5.259	0.068			
Gender	0.791	0.332–1.885	0.597			
Pathological type	0.805	0.298–2.176	0.669			
B symptom	1.114	0.498–2.493	0.792			
Elevated LDH	2.703	1.079–6.772	0.034			
ECOG > 1	0.040	0.000–7.141	0.224			
Advanced stage	2.126	0.635–7.115	0.221			
IPI score	0.944	0.396–2.251	0.897			
Ki-67 > 75%	1.210	0.505–2.898	0.669			
Extranodal involvement > 1	1.056	0.456–2.444	0.899			
TET2 mutation	1.646	0.689–3.937	0.262			
TP53 mutation	1.083	0.405–2.895	0.874			
TET2/TP53 mutation	3.535	1.546–8.083	0.027	3.573	1.069–11.941	0.039

*OS* Overall survival, *HR* Hazard ratio, *CI* Confidence interval, *LDH* Lactate dehydrogenase, *ECOG* Eastern Cooperative Oncology Group, *IPI* International Prognostic Index

^a^Factors with P < 0.10 in the univariate analyses were subjected to multivariate analysis afterwards, including age, elevated *LDH*, and *TET2/TP53* mutation

^b^Forward stepwise Cox proportional-hazard modeling was used in multivariate analysis of risk factors

**Fig. 4:**
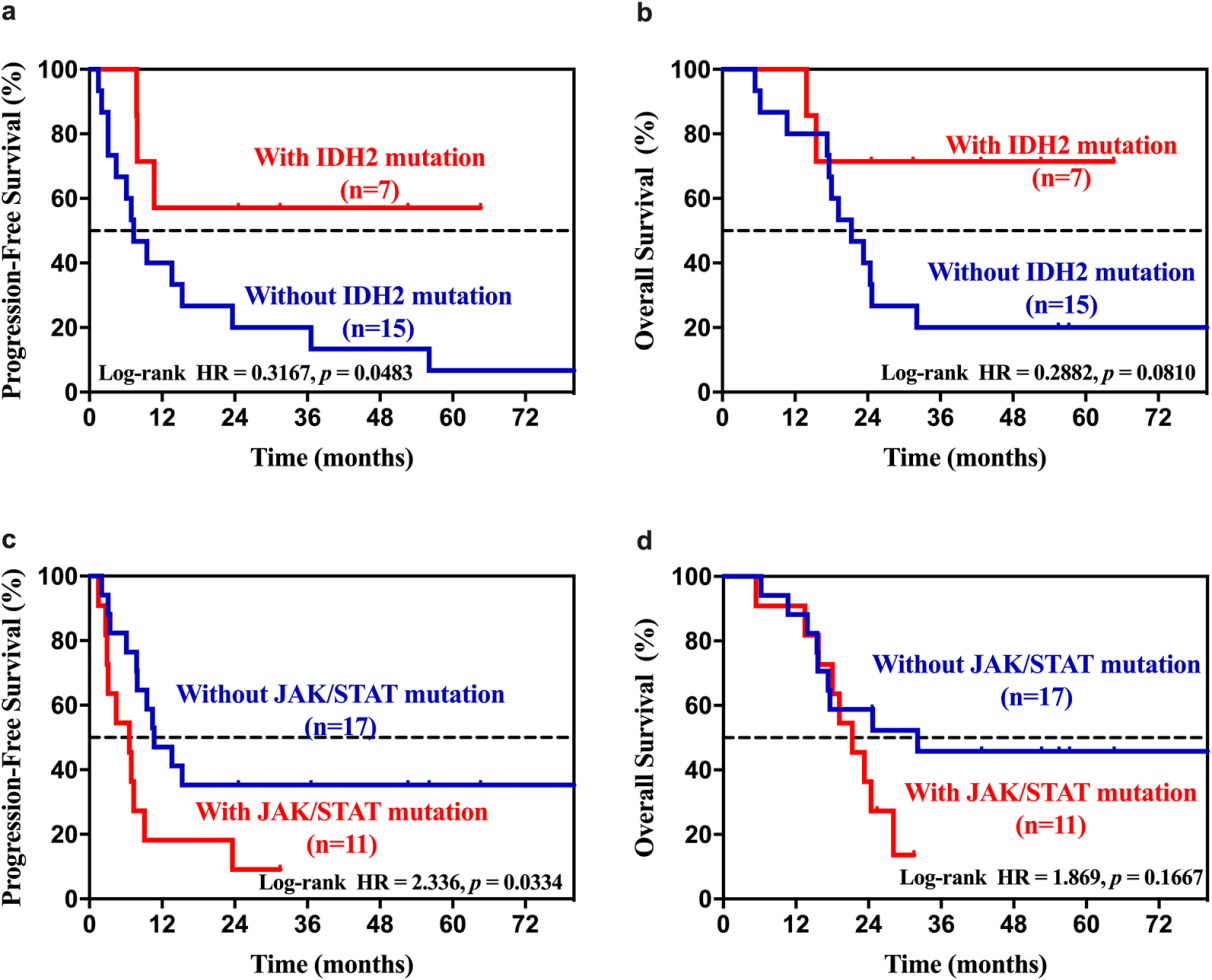
Kaplan–Meier curves for univariate analysis in AITL patients. **a**, **b** *IDH2* mutation. **c**, **d** JAK/STAT pathway mutation

Epigenetic/chromatin modifiers were reported to be frequently mutated in AITL patients. Given that our study cohort was comprised mainly AITL patients (28/46), we further analyzed survival in this subtype of patients. After we excluded 7 patients who have received epigenetic therapy, AITL patients with *IDH2* mutations had a superior PFS compared to patients without mutations (HR 0.3167, 95% CI 0.1227–0.8177, *P* = 0.0483, Fig. 4a). In addition, we also found a significant correlation between the JAK/STAT pathway mutations and the PFS of ATIL patients. The occurrence of JAK/STAT pathway mutations in AITL patients was shown to confer an inferior PFS (HR 2.366, 95% CI 0.9130–6.129, *P* = 0.0334, Fig. 4c).

## Discussion

PTCL patient outcomes are generally dismal and their long-term survival prospects have not been improved by the existing treatment regimens or even novel drugs [14]. Therefore, a better understanding of the pathogenesis and biology of PTCL is needed to help develop novel therapeutic approaches and improve patient outcomes. By analyzing a series of PTCL cases, we have refined the information related to previously reported genetic alterations and oncogenic pathways. Furthermore, we explored the prognostic impact of these mutations in PTCL patients.

In our study, we found that the most common mutations occurred in epigenetic/chromatin remodeling, followed by T-cell activation, DNA repair/TP53 pathway, the RTK pathway, the PI3K/AKT pathway and the JAK/STAT signaling pathway. Frequent alterations in epigenetic modifiers, including *TET2*, *DNMT3A* and *IDH2*, the TCR signaling pathway, and the JAK/STAT pathway, have been reported previously [4, 6, 15–17]. Overall, our sequencing results showed that different subtypes of PTCLs have their unique characteristics with a high level of heterogeneity among them. According to Heavican and other researchers’ studies, T follicular helper (TFH) cells could be identified as the cell of origin of AITLs [18, 19]. AITL has been characterized by recurrent mutations in genes of epigenome regulators (*TET2*, *DNMT3A*, and *IDH2*^R172^) as well as the proximal TCR and costimulatory signaling pathways [6]. Inactivating *TET2* mutations have been found in up to 85% of AITL patients [8]. In accordance with previous studies, the AITL patients in our cohort were more likely to harbor mutations in genes of epigenetic modifiers and T-cell activation pathway (*TET2* 85%, *RHOA* 64% and *IDH2* 27%). We also found that the frequency of TET2 mutations is significantly higher in patients with positive B symptom or elevated LDH level, as confirmed in the study by Zhifang Xu’s team [20]. High LDH level and positive B symptom is known as an indicator of tumor burden and plays a crucial role in tumor maintenance [21].

Most of the *RHOA* mutations (56/68, 82%) detected in our study were proved to be RHOA^G17V^ variants (c.50G > T), which results in a glycine to valine substitution at residue 17 of the RHOA protein [22]. Furthermore, 95% of AITL patients with *RHOA* mutations also harbored *TET2* mutations. Cortes et al. reported that *RHOA*^*G17V*^ expression combined with *TET2* mutation results in the development of AITL in mice, suggesting the existence of crosstalk between these mutations in AITL [9]. However, there was no combinational effect of *TET2* and *RHOA* mutations on patient survival in this study. We found that the co-occurrence of *TET2* and *IDH2* mutations conferred a better PFS, compared with *TET2* alone mutation, which also highlighting the importance of epigenetic modifications in PTCL patients.

We also detected *TET2* mutations in PTCL-NOS patients, but at a lower frequency than the other PTCL entities, which may due to the TFH-like subtype of PTCL-NOS. Apart from *TET2* alterations, mutations in PTCL-NOS patients were predominantly observed in *PCLO* and *TP53.* Regarding genomic alterations, *TP53* mutations were detected at a much lower frequency in PTCLs compared with B-cell lymphoma [23] and have not been extensively investigated. In our study, we detected frequent *TP53* mutations (15%), which occurred mainly in ALK- ALCL and PTCL-NOS patients. A recent genomic study showed that *TP53* mutations were associated with PTCL-GATA3 [24]. *TP53* alterations are reported more commonly in ALK- ALCL patients than in ALK + patients and may contribute to a more aggressive disease course [25]. A study by Vasmatzis et al. also suggested that a series of genetic mutations and epigenetic abnormalities may conduce destruction of p53-associated tumor suppressor function in PTCL [26].

Of note, we observed frequent mutations in *PCLO* (23%), which is reported to participate in modulating neurotransmitter release and critical for the recycling and maintenance of synaptic vesicles [27]. Recurrent mutations in *PCLO* were also discovered in diffuse large B-cell lymphoma [28] and several solid tumors, including glioblastoma, liver cancers, and stomach adenocarcinoma [29–32]. Qiu et al. have reported that *PCLO* mutations could precisely predict etoposide sensitivity in small cell lung cancer [29]. *PCLO* alterations have not yet been reported in PTCL and further researches are warranted to clarify the influence of *PCLO* mutations in PTCL.

We found no difference in the outcome of PTCL patients with any individual mutation. In AITL patients, the only individual mutation which confers a significant inferior survival was *IDH2*. The adverse impact of *TET2*, *RHOA*, and *DNMT3A* mutations in AITL reported by other groups [2, 8] were not observed in our study. In contrast to our findings, previous studies in PTCL-NOS showed that mutations in histone methyltransferase genes (*KDM6A*, *MLL*, or *MLL2*) were corelated with an adverse clinical outcome [17]. The reasons for this discrepancy are still undetermined but may be due to a relatively small patient cohort size. In our study, the combination of *TET2* and *TP53* mutations provided a much better prognostic capacity and *TET2/TP53* mutation was proved to be an independent prognostic factor for worse overall survival. A recent study revealed a functional interaction between p53 and TET2 during anti-cancer treatment [33]. In p53 knockout cells, TET2 accumulates in the nucleus and protects the genome from DNA damage, which promotes cell proliferation and ultimately leads to chemotherapy resistance.

The standard treatment for PTCL remains undefined. In spite of their significant differences in clinical manifestation and pathologic appearance, the most common entities of PTCL, including AITL, PTCL-NOS and ALCL (ALK positive and negative), tend to be treated similarly [34]. And approximately 85% PTCL patients received CHOP or CHOP-like regimens within frontline treatment [14]. In our study, we analyzed the efficacy and outcome of CHOP-like therapy in PTCL patients. Our results showed that the overall response rate (ORR) to CHOP-like regimens was 65.2% and the CR rate was 54.3%, which is consistent with previous research data [35–37]. Several prospective and retrospective autologous stem cell transplant (ASCT) researches have revealed an improved outcome in PTCL patients with an OS rate of 60–70% [38, 39]. We also observed superior OS among patients treated ASCT consolidation therapy, compared with those receiving CHOP-like therapy alone.

The limitations of our study should be noted. We identified an association of *TET2* and *TP53* mutations with inferior survival in PTCL; however, the number of patients recruited in our cohorts was relatively small. Thus, our results should be interpreted with caution and further prospective studies of *TET2/TP53* mutations and PTCL are warranted. The mechanisms underlying the association of *TET2* and *TP53* mutations with an adverse prognosis for PTCL patients are still unclear. In future research, a large cohort of PTCL patients will be enrolled to validate the initial findings of this study. We will also conduct further investigations to determine the transcriptome profiles of *TET2/TP53* mutations in PTCLs to gain additional insights into their consequences.

## Conclusion

Our findings suggest that heterogeneous gene mutations occur in PTCL patients. *TET2*/*IDH2* co-mutation conferred a superior PFS, compared with *TET2* mutation, and *TET2/TP53* mutations are associated with an adverse prognosis. The JAK/STAT pathway had a negative impact on survival outcomes in AITL patients, thus implicating this as a potential target for this malignant subtype.

## Supplementary Information

**Additional file 1: Table S1.** Gene list.

**Additional file 2: Table S2.** Somatic mutations detected in patients with PTCL.

**Additional file 3: Figure S1.** (a) The distribution of gene mutations in different histological subtypes of PTCL: The bars indicate mutation frequencies in each subtype. (b) The distribution of mutated functional groups in different histological subtypes. Gene mutation function proportion of TET2 (c) and TP53 (d) in newly diagnosed PTCL patients.

**Additional file 4: Table S3.** Patients' characteristics based on gene mutational status.

**Additional file 5: Table S4.** Impact of mutations on the efficacy of CHOP‐like chemotherapy.

**Additional file 6: Figure S2.** Kaplan–Meier curves for univariate analysis. Stratified by (a) ASCT, (b) TET2 mutation, (c) IDH2 mutation. (d) TET2/IDH2 co-mutation vs TET2 alone mutation or IDH2 alone mutation. (e) TET2/RHOA co-mutation vs TET2 alone mutation or RHOA alone mutation. (f) TET2/DNMT3A co-mutation vs TET2 alone mutation or DNMT3A alone mutation.

## Data Availability

All relevant data of the study are included within the article and its additional files. Further inquiries of the original data can be directed to the corresponding authors.

## Abbreviations

PTCL: Peripheral T-cell lymphoma
LDH: Lactate dehydrogenase
AITL: Angioimmunoblastic T-cell lymphoma
NHL: Non-Hodgkin lymphoma
TET2: Ten-eleven translocation 2
IDH1/2: Isocitrate dehydrogenase 1/2
DNMT3A: DNA methyltransferase 3A
TCR: T-cell receptor
gDNA: Genomic DNA
FFPE: Formalin-fixed, paraffin-embedded
NGS: Next-generation sequencing
SNVs: Single nucleotide variants
Indels: Insertions and deletions
OS: Overall survival
PFS: Progression-free survival
ALK: Anaplastic lymphoma kinase
ALCL: Anaplastic large cell lymphoma
PTCL-NOS: Peripheral T-cell lymphoma, not otherwise specified
CDS-Indel: Coding sequence insertion-deletion
CHOP: Cyclophosphamide, doxorubicin, vincristine, prednisone
ASCT: Autologous stem cell transplant
OR: Overall response
CR: Complete response
PR: Partial response
WT: Wild-type
HR: Hazard ratio
CI: Confidence interval
TFH: T follicular helper
ORR: Overall response rate
