# Supplementary material for: Correlation of mutational landscape and survival outcome of peripheral T-cell lymphomas
Source: Exp Hematol Oncol. 2021 Feb 5;10:9. doi: 10.1186/s40164-021-00200-x (PMC7866778; doi:10.1186/s40164-021-00200-x)
Supplement: Supplementary file 3 — Additional file 3: Figure S1. (a) The distribution of gene mutations in different histological subtypes of PTCL: The bars indicate mutation frequencies in each subtype. (b) The distribution of mutated functional groups in different histological subtypes. Gene mutation function proportion of TET2 (c) and TP53 (d) in newly diagnosed PTCL patients. [file 40164_2021_200_MOESM3_ESM.pdf]

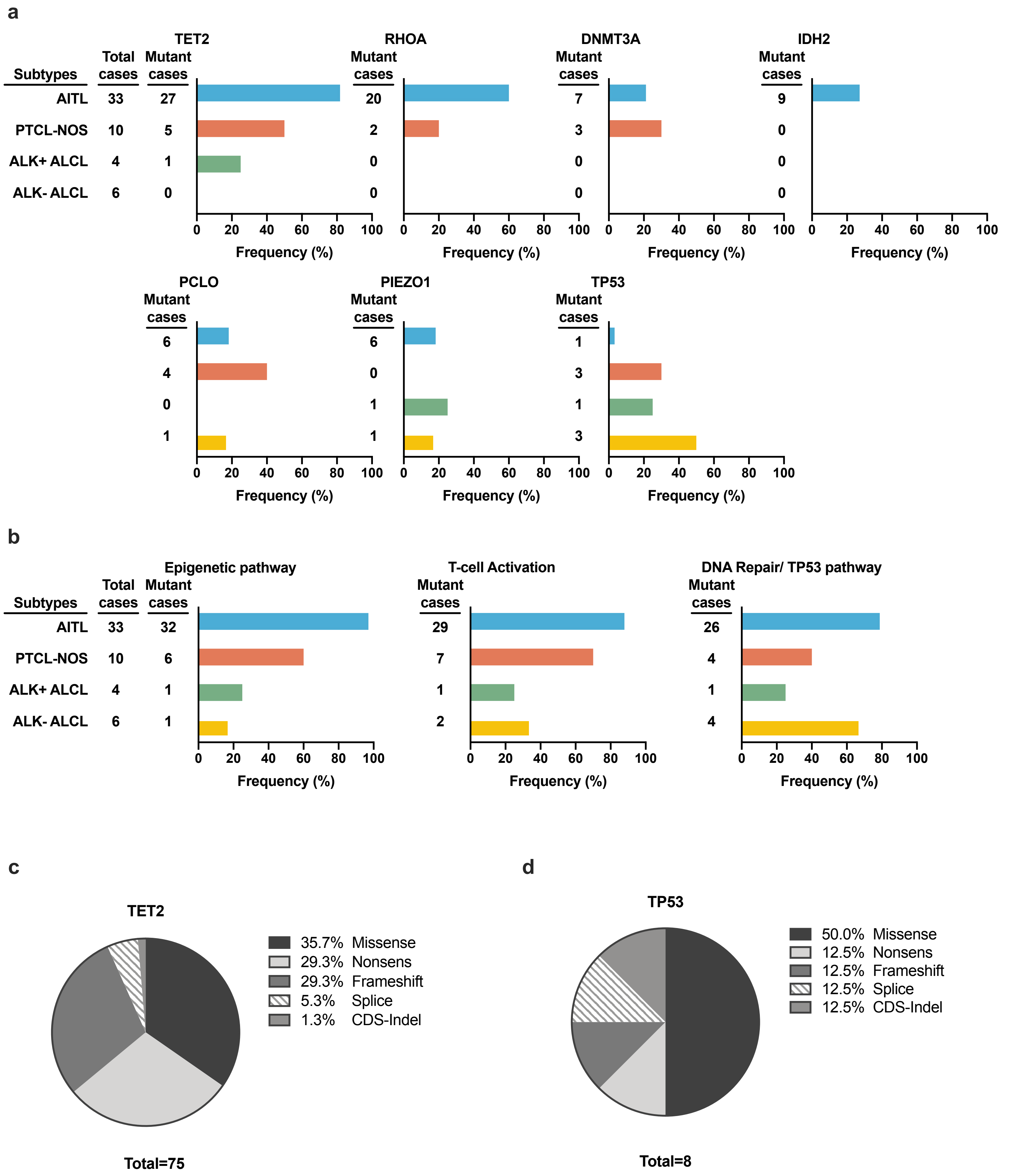

Figure S1. (a) The distribution of gene mutations in different histological subtypes of PTCL: The bars indicate mutation frequencies in each subtype. (b) The distribution of mutated functional groups in different histological subtypes. Gene mutation function proportion of TET2 (c) and TP53 (d) in newly diagnosed PTCL patients.
