# Supplementary material for: Correlation of mutational landscape and survival outcome of peripheral T-cell lymphomas
Source: Exp Hematol Oncol. 2021 Feb 5;10:9. doi: 10.1186/s40164-021-00200-x (PMC7866778; doi:10.1186/s40164-021-00200-x)
Supplement: Supplementary file 6 — Additional file 6: Figure S2. Kaplan–Meier curves for univariate analysis. Stratified by (a) ASCT, (b) TET2 mutation, (c) IDH2 mutation. (d) TET2/IDH2 co-mutation vs TET2 alone mutation or IDH2 alone mutation. (e) TET2/RHOA co-mutation vs TET2 alone mutation or RHOA alone mutation. (f) TET2/DNMT3A co-mutation vs TET2 alone mutation or DNMT3A alone mutation. [file 40164_2021_200_MOESM6_ESM.pdf]

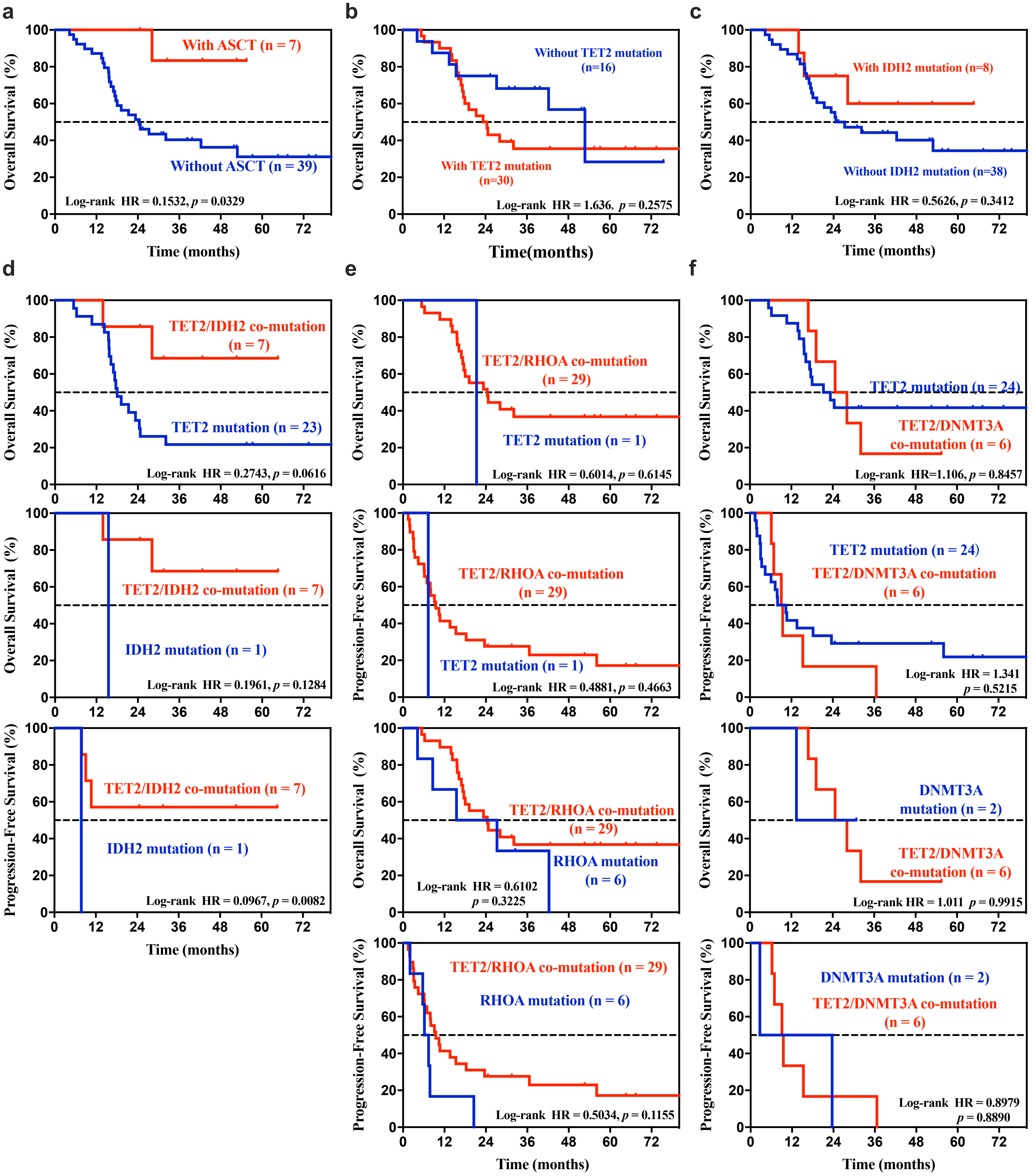

Figure S2. Kaplan-Meier curves for univariate analysis. Stratified by (a) ASCT, (b) TET2 mutation, (c) IDH2 mutation. (d) TET2/IDH2 co-mutation vs TET2 alone mutation or IDH2 alone mutation. (e) TET2/RHOA co-mutation vs TET2 alone mutation or RHOA alone mutation. (f) TET2/DNMT3A co-mutation vs TET2 alone mutation or DNMT3A alone mutation.
